# Effects of Leonardite Amendments on Vineyard Calcareous Soil Fertility, Vine Nutrition and Grape Quality

**DOI:** 10.3390/plants11030356

**Published:** 2022-01-28

**Authors:** Miguel Ángel Olego, Mateo Cuesta Lasso, Miguel Javier Quiroga, Fernando Visconti, Roberto López, Enrique Garzón-Jimeno

**Affiliations:** 1 Research Institute of Vine and Wine, Universidad de León, Avenida de Portugal, 41, CP 24071 León, Spain; mcuel@unileon.es (M.C.L.); germqm@unileon.es (M.J.Q.); jegarj@unileon.es (E.G.-J.); 2 Centro para el Desarrollo de la Agricultura Sostenible, Instituto Valenciano de Investigaciones Agrarias, Carretera CV-315, km 10.7, CP 46113 Valencia, Spain; visconti_fer@gva.es; 3 Department of Applied Chemistry and Physics, Faculty of Biology and Environmental Sciences, Campus de Vegazana, Universidad de León, CP 24071 León, Spain; rlopg@unileon.es

**Keywords:** ferric chlorosis, grapevine, leonardite, potassium, soil organic matter

## Abstract

Vineyard calcareous soils are usually low in organic matter, which makes them prone to physical, chemical, and biological degradation. Besides, these soils are also usually poor in various nutrients in plant-available form, e.g., iron. To make up for this lack of soil fertility, on the one hand, manures, and on the other, iron chelates are usually used. However, the soil application of these materials is not free from problems, and other amendments based on leonardites could be advantageously used as an alternative. Therefore, two organic amendments, one leonardite alone (1 Mg/ha), and the other leonardite (1 Mg/ha) plus ferrous sulphate heptahydrate (0.5 Mg/ha), were tested for three years in a commercial vineyard calcareous plot under Mediterranean climate. The effects of these amendments on soil fertility, plant nutrient contents, and berry quality were studied against a control of bare soil by means of a fully randomized trial with three repetitions per treatment. Soil organic matter (SOM) increased as a consequence of both leonardite treatments, but much more than expected on the basis of a simple mass transfer from the amendments. With the ferrous-sulphate-heptahydrate-supplemented leonardite, the increase in SOM was noticeably higher. This is explained on the basis of nutrient quantity and intensity-pH-related effects, which increased soil nutrient plant-availability and presumably enhanced vine root growth. In response to the higher plant availability of nutrients, the petiole nutrient concentrations were observed to increase under the leonardite treatments. However, only a trend to increase potassium in petioles and in grape must, linked to a decrease of grape must pH, was observed in harvest quality under the leonardite treatments. Leonardite and adequately supplemented leonardite seem to have potential for increasing SOM contents and nutrient plant-availability, thus improving the soil fertility of vineyard calcareous soils.

## 1. Introduction

There are three core soil properties (texture, mineralogy, and soil organic matter (SOM)) that constitute the natural capital of soils [1]. Soil texture and mineralogy are inherent properties of soil, inherited from bedrock, and slowly evolve along years, whilst SOM levels dramatically change with land use and management [1]. Despite the small percentage that SOM represents in most soils [2], it improves the buffering and cation exchange capacity in soils, having a high heavy metal adsorption and complexing capacity. A decrease of SOM can lead to the drastic impairment of soil physical and chemical properties, with negative impacts on soil nutrient cycling mechanisms [3] as well as on crop production. Therefore, the quality of soils determines agricultural sustainability and environmental quality [4], but the impact of SOM and its humic substances originated by humification on crop productivity is quite complex because it affects a range of soil properties, not just a single one [5]. These reasons reflect the importance of SOM as a soil component, which can be summarised according to its important influence on soil quality.

The SOM benefits to grapevines are indirect: influences water retention and permeability as well as aggregate structure, nutrient availability, and buffering capacity [6]. Moreover, in order to maintain profitable grape production sustainability in many situations, the use of external inputs, such as chemical fertilizers, should be lowered and SOM contents increased [7]. For most vineyard soils, the ideal SOM content is in the range 1–3% [8]. In this sense, White and Krstic [9] provide optimal values for soil organic carbon based on soil textural classes. However, the right amount of SOM in a vineyard is a function of land management decisions like the amount and weight of traffic or the presence or absence of cover crops [10]. The soil management of Mediterranean vineyards, despite their low organic matter content, usually consists in continuous tillage maintaining bare soils [11]. Such intensive working of vineyard soils can lead to soil degradation, with loss of soil fertility, acceleration of soil erosion, SOM mineralization, and hence increased ${\mathrm{CO}}_{2}$ emission [3] and nutrient loss [11]. Specific vineyard soil properties like coarse textural classes can lead to higher SOM losses. Thus, the maintenance of appropriate SOM levels becomes essential to sustain the Mediterranean viticulture, as it is also critical to maintain high agriculture productivity [12]. However, although a continuous supply of organic material must be met to maintain an appropriate level of SOM in vineyard soils, we must focus on the principles of efficient and ecologically sound management of SOM with organic fertilisers.

Worldwide, manures have been spread on arable soils over the years [13,14] as an important supply of organic matter, useful microorganisms, and plant nutrients [15]. However, the role of manure in soil fertility and SOM maintenance involves some potential environmental drawbacks, such as water pollution as a consequence of high surface nutrient concentrations (N and P) [16], or increases of toxic heavy metals in soil surface layers [15,17,18]. This could be of high prominence for Cu as it is widely used as an additive in most animal feeds [18]. An inadequate manure management can lead to dispersion of clay colloids, fostered by $K^{+}$, ${\mathrm{Na}}^{+}$, and ${\mathrm{NH}}_{4}^{+}$ accumulation, which is associated with large applications of manure [19], and at the same time a decrease of soil stability in vineyards [20]. Additionally, in the last 30 years, the occurrence of antibiotics and hormones, both in raw or treated livestock manures (swine, cattle, poultry and horse), has been widely reported [14]. Moreover, Gravert et al. [21] found steroids in agricultural soils amended with cattle manure, whereas Kjær et al. [22] warned about the contamination risk to the aquatic environment due to the leaching of steroid hormones, potential endocrine disruptors, from swine manure-treated fields. Finally, livestock manure may also contain large numbers of pathogenic microorganisms, which can potentially pose risks to human health [15].

Therefore, to maintain a sustainable agriculture practice meeting environmental regulations, alternative organic fertilizer sources should be found. One alternative that may be used as a substitute of manures is leonardite. Originating from an oxidation product of lignite related to subsurface mining or as sediments enriched with humic compounds [23], leonardite is a concentrated form of humic and fulvic acids [24], and its application has been shown to improve crop nutrient uptake [23,25]. Because humic acids along with fulvic acids are essential components of soil organic matter, playing a critical role in improving soil properties [26], it is expected that the use of leonardite directly and leonardite-derived humic substances as soil amendments and plant stimulants will improve the physicochemical and biological aspects of soil, promoting plant yield and quality [25,27,28]. Despite the above, previous research has focused on identifying and evaluating the effects of leonardite on soil trace element mobility [29,30,31,32], soil microbiome structure and functionality [24,28,30], stability of soil aggregates [33], as well as crop tolerance to soil drought [23] and salinity [34]. However, less research has been performed on how this organic material affects grapevine nutrition and harvest 5uality.

Besides the lack of SOM in most vineyard soils, there is also the issue of iron (Fe) deficiency. This is a disorder affecting crops in many areas of the world, mainly associated with high pH, calcareous soils that make most of soil Fe unavailable for plants [35]. In the case of high value crops like vineyards, which are the grape source for expensive wines, the prevention or correction of Fe deficiency is usually solved by applying costly fertilizers, such as synthetic Fe(III) chelates [36]. An alternative to this practise is the use of fertilizers based on inorganic Fe-compounds which include some soluble iron species, such as Fe(II) (e.g., ${\mathrm{FeSO}}_{4}\cdot 7H_{2}O$ (ferrous sulphate heptahydrate)). However, this technique seems to become quite inefficient, especially in high pH soils, due to the rapid transformation of most of the applied Fe into highly insoluble compounds [35] when they are applied alone. Thus, another aim of this work was to investigate whether the use of leonardites alone and mixed with ferrous sulphate heptahydrate (FSH) could serve to mitigate Fe deficiency on grapevines.

For the sake of completion, the objectives of this research were to assess the effects of the use of (1) leonardite and (2) leonardite mixed with FSH on several properties, including the SOM, of calcareous vineyard soils and on the concentration of mineral nutrients in petiole tissues, as well as on harvest quality characteristics.

## 2. Results

### 2.1. Soil Properties

**Figure 1:**
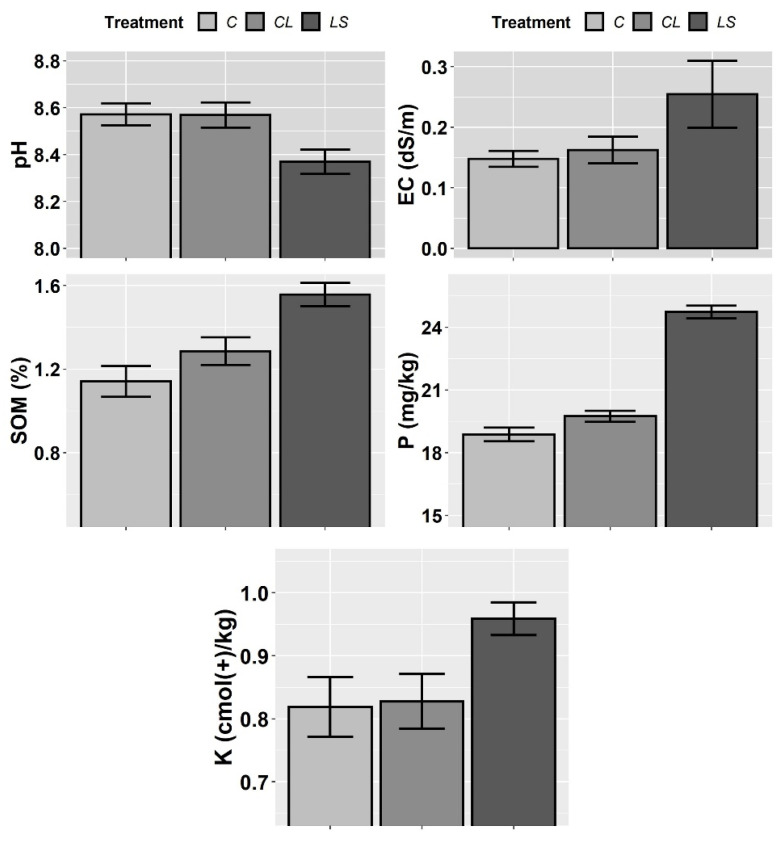
Mean values of the soil parameters pH, electrical conductivity (EC), soil organic matter (SOM), phosphorus (P) and potassium (K) for each treatment. Treatments: control (C), control of leonardite (CL), and leonardite mixed with ferrous sulphate heptahydrate (LS). Error bars reflect the SE of the mean (±1 SE mean).

**Figure 2:**
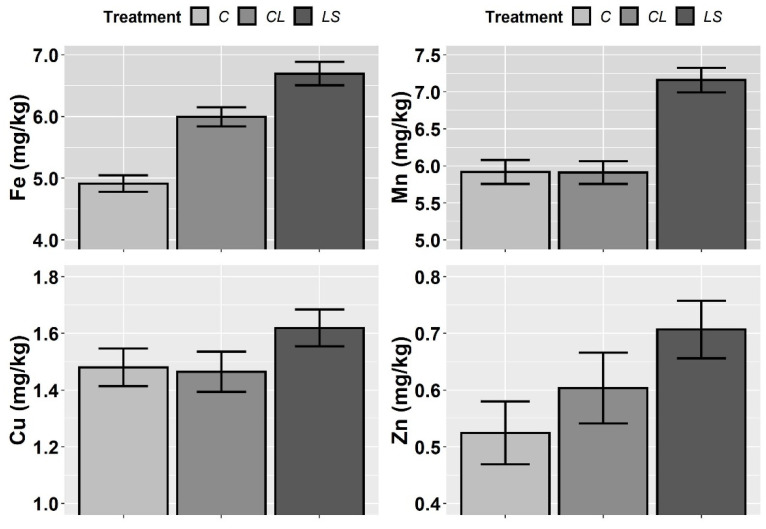
Mean values of the soil parameters iron (Fe), manganese (Mn), copper (Cu) and zinc (Zn) for each treatment. Treatments: control (C), control of leonardite (CL), and leonardite mixed with ferrous sulphate heptahydrate (LS). Error bars reflect the SE of the mean (±1 SE mean).

**Figure 3:**
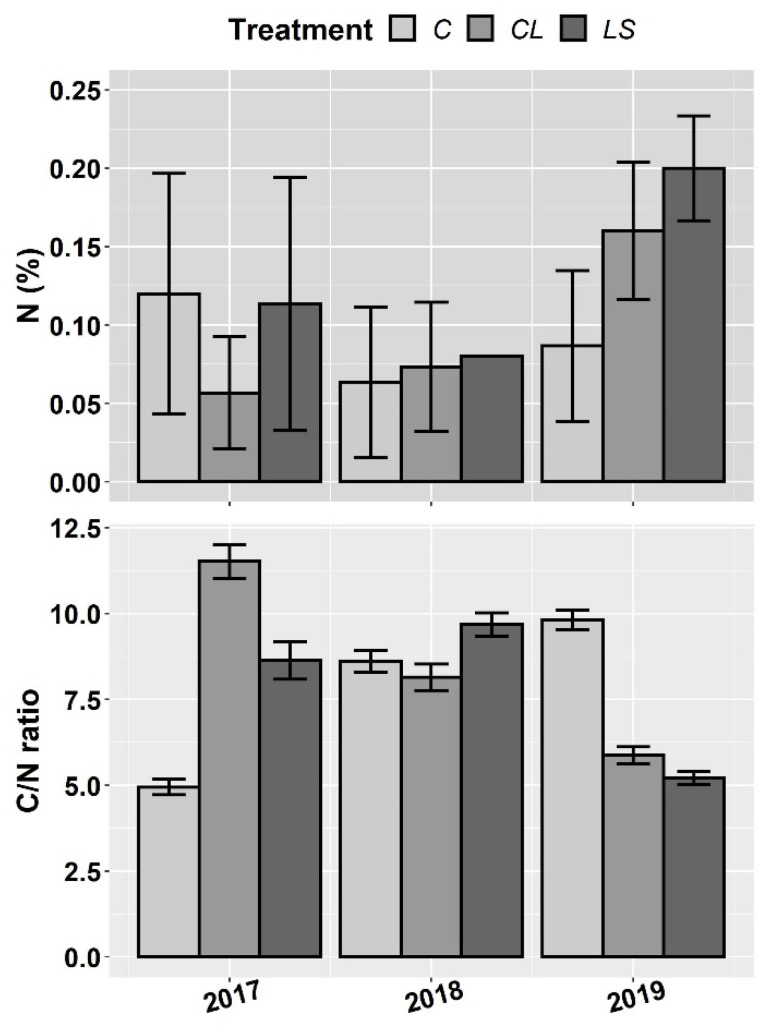
Mean values of the soil parameters nitrogen (N) and C/N ratio for each treatment and year. Treatments: control (C), control of leonardite (CL), and leonardite mixed with ferrous sulphate heptahydrate (LS). Error bars reflect the SE of the mean (±1 SE mean).

**Figure 4:**
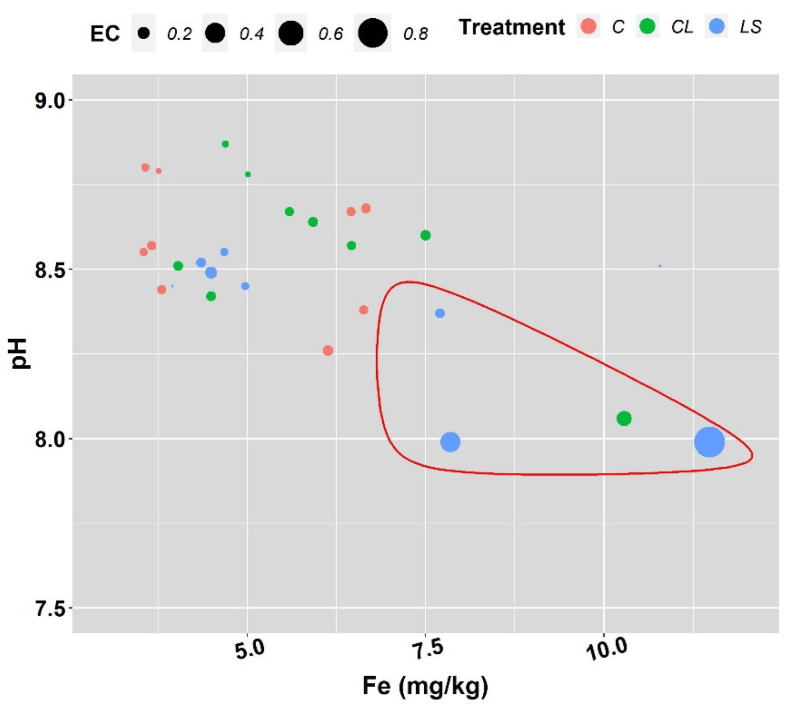
Influence of treatments on soil pH, electrical conductivity (EC in dS/m) and iron (Fe) plant availability. Cases where plant-available Fe was ≥7.5 mg/kg and soil pH was ≤8.5 are highlighted with a red line. Treatments: control (C), control of leonardite (CL), and leonardite mixed with ferrous sulphate heptahydrate (LS).

In Figure 1, Figure 2 and Figure 3, the means and standard errors of the soil properties for the control and treatments the three years of monitoring are shown. Where no significant interactions between treatment (T) and year of sampling (Y) were found, the joint three-year means and standard errors are shown. In this first visual inspection of data some differences between treatments are worth commenting. Remarkably, the control of leonardite (CL) and leonardite mixed with ferrous sulphate heptahydrate (LS) stand out for its ability to increase SOM, and it seems that more the LS than the CL. Besides, the LS highlights for its ability to decrease soil pH at the same time that it enhances EC, P, and K (Figure 1), and additionally, the micronutrients Fe, Mn, Cu, and Zn (Figure 2). In Figure 4, it can be seen that LS is linked to lower soil pH and higher EC and Fe.

According to the mixed analyses of variance (ANOVAs), a significant effect of the treatments was indeed revealed for soil pH, SOM, Mn, and Zn. Moreover, the effect of the year of sampling on each of these soil properties, as well as on soil N, Fe and Cu, was also observed to be significant. Besides, the effects of the treatments significantly changed in magnitude or direction with the year of sampling for both soil N and C/N ratio, as revealed by the significant interaction between both factors (T × Y) in these soil properties (Table 1). In accordance with the specific hypotheses that were tested in the planned contrasts, i.e., only where the likelihood ratio of mixed ANOVAs was statistically significant, the following was found (Table 2). Firstly, soil pH significantly decreased from the control to the treatments, whereas SOM and Zn increased (contrast 1). Secondly, soil pH again significantly decreased from one treatment (CL) to the other (LS), and Mn significantly increased from CL to LS (contrast 2). No more significant changes were observed in SOM and Zn from one treatment to the other.

### 2.2. Petiole Nutrients

**Figure 5:**
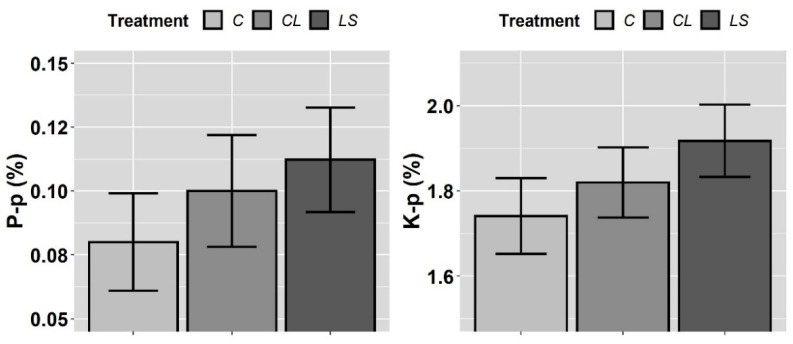
Mean values of the petiole nutrient contents of phosphorus (P-p) and potassium (K-p) for each treatment. Treatments: control (C), control of leonardite (CL), and leonardite mixed with ferrous sulphate heptahydrate (LS). Error bars reflect the SE of the mean (±1 SE mean).

**Figure 6:**
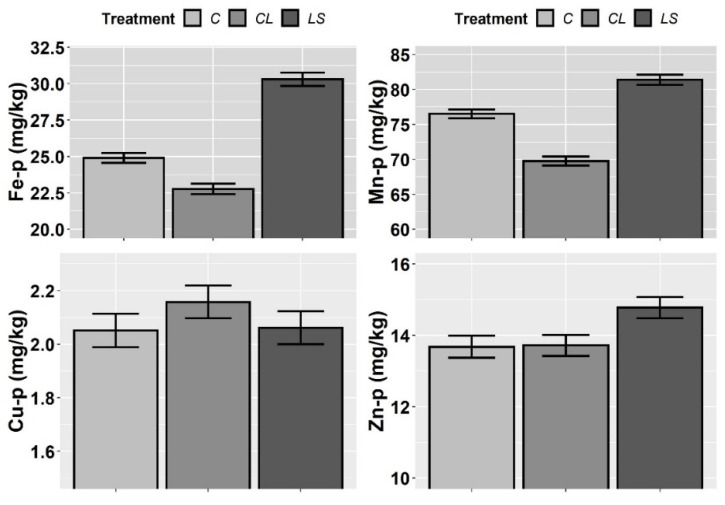
Mean values of the petiole nutrient contents of iron (Fe-p), manganese (Mn-p), copper (Cu-p) and zinc (Zn-p) for each treatment. Treatments: control (C), control of leonardite (CL), and leonardite mixed with ferrous sulphate heptahydrate (LS). Error bars reflect the SE of the mean (±1 SE mean).

In Figure 5 and Figure 6, the means and standard errors of the P, K, Fe, Mn, Cu, and Zn nutrient contents in the petioles for the control and treatments during three years of monitoring are shown. Because there were no significant interactions between T and Y for any element (Table 3), the joint three-year means and standard errors are shown for all. In this first visual inspection of data, there are differences among treatments that are worth commenting on. Firstly, it seems that both treatments (CL and LS) increase P-p and K-p with regard to the control while, additionally, LS seems to specifically increase Fe-p, Mn-p, and Zn-p.

However, according to the mixed ANOVAs, a significant effect of the treatments was found only on P-p. Moreover, the year of sampling had a more significant effect on P-p, and on K-p, Fe-p, Cu-p, and Mn-p, than any of the treatments (Table 3). In accordance with the specific hypotheses that were tested in the planned contrasts i.e., only where the likelihood ratio of the mixed ANOVAs was statistically significant, the P-p level significantly increased from the control to both CL and LS (contrast 1), whereas no differences were found between CL and LS (contrast 2) (Table 4).

### 2.3. Harvest Parameters

**Figure 7:**
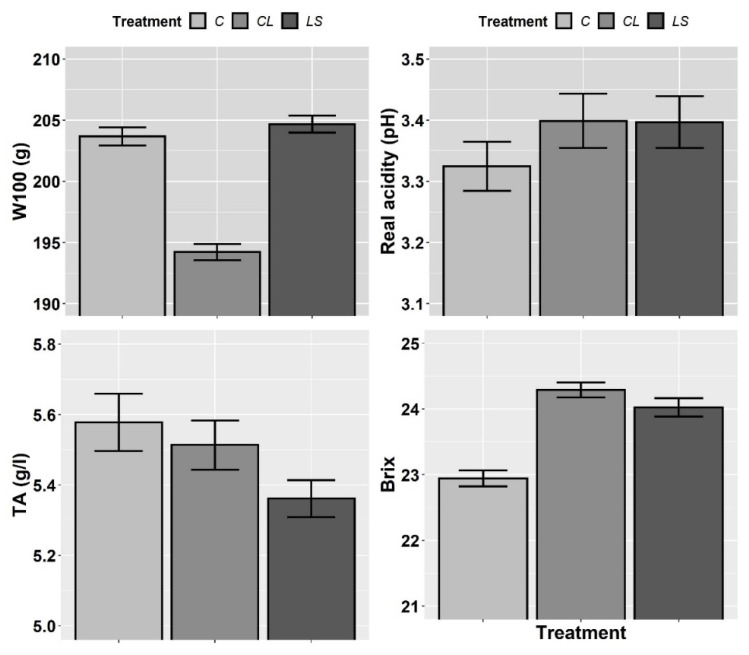
Mean values of the harvest parameters (weight of 100 grape berries (W100), real acidity (pH), titratable acidity (TA) and Brix degrees (Brix)) for each treatment. Treatments: control (C), control of leonardite (CL), and leonardite mixed with ferrous sulphate heptahydrate (LS). Error bars reflect the SE of the mean (±1 SE mean).

**Figure 8:**
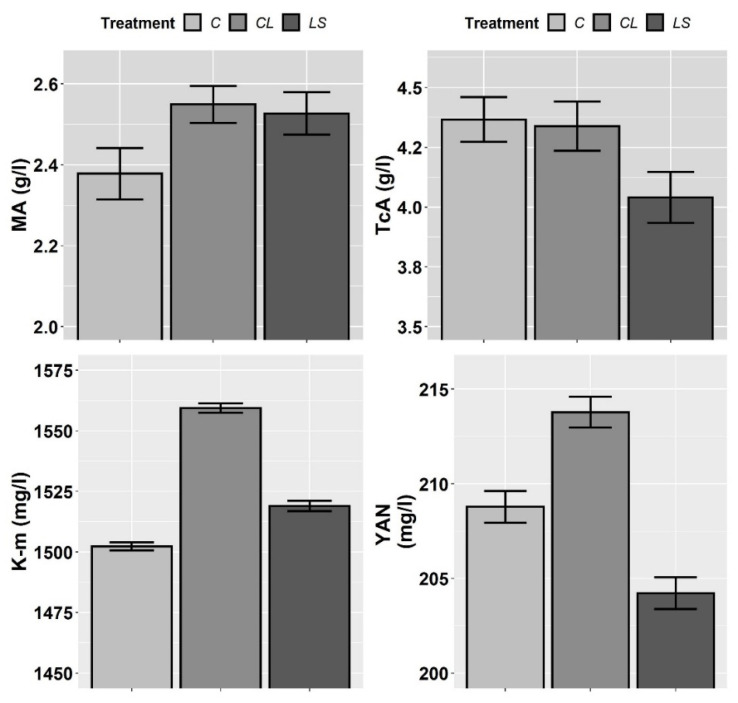
Mean values of the harvest parameters (malic acid (MA), tartaric acid (TcA), must potassium levels (K-m) and yeast assimilable nitrogen (YAN)) for each treatment. Treatments: control (C), control of leonardite (CL), and leonardite mixed with ferrous sulphate heptahydrate (LS). Error bars reflect the SE of the mean (±1 SE mean).

In Figure 7 and Figure 8, the means and standard errors of the harvest quality parameters (W100, real acidity (pH), TA, Brix, MA, TcA, K, and YAN) for the control and treatments during three years of monitoring are shown. As in the case of most soil parameters and all petiole nutrients, no interaction between T and Y was found (Table 5). Therefore, the joint three-year means and standard errors are shown. In this first visual inspection of data, it seems that both the CL and LS treatments increase real acidity, Brix, and K-m levels and decrease TA regarding the control.

However, according to the mixed ANOVAs, a significant effect of the treatments only was found on real acidity. What is more, similarly to what happened with the nutrients determined in the petioles, the effect of the year of sampling was found to be more significant on real acidity than the treatments, and the effect of the year was also found significant on W100, K, and YAN (vintage effect) (Table 5). In accordance with the specific hypotheses that were tested in the planned contrasts, both the CL and LS treatments significantly increased real acidity regarding the control (contrast 1), whereas no differences were found between CL and LS (contrast 2) (Table 6).

## 3. Discussion

According to the amendments’ rates and compositions, as well as the method of application, SOM increased each year in 0.064% and 0.067% following the addition at the onset of crop season of, respectively, the leonardite-alone amendment and the leonardite-plus-FSH one (Table 7). However, at the end of the season, SOM was found to be in each treatment, respectively, 0.10% and 0.45% higher than the control on average. Therefore, most of the observed increase in SOM cannot be due to a simple transfer of organic matter mass from the amendments to the soil environment. Besides, the leonardite amendments did not only work as a supply of organic matter to the soil, but also of several nutrients (Table 7). This can make a difference for plant nutrition regarding the control in a soil as nutrient-depleted as this, to which no other fertilizers were applied. Although this difference for plant nutrition could only be confirmed in the plant on a statistical basis for P-p, interesting trends for higher petiole nutrient contents could be observed for K-p, Fe-p, Mn-p, and Zn-p, overall, with the leonardite-plus-FSH amendment.

In agricultural systems, in general, 70% of SOM can be traced back to plant roots and 30% to the aerial plant organs, including the incorporated crop residues [37,38]. Since in the study site weed growing on the lanes between plants is systematically prevented, and since vine crop residues are not incorporated, the most important source of organic matter to the soil are the vine roots. Therefore, it can be hypothesized that the higher nutrient content in the amended soil area fostered root growth in that soil part, and the subsequent organic rhizodeposition effectively contributed to the observed SOM increase by providing fresh organic material to the soil organisms.

Such an increase in SOM as a consequence of the addition of leonardite was also found in other works [24,27]. Besides, the higher values of extracted Fe and Zn obtained in subplots fertilized with leonardites agree with Maqueda et al. [39], even though those obtained for Cu and Mn are not in agreement with them.

In the present work, however, one leonardite treatment was supplemented with an extra source of nutrients, i.e., the FSH, and this supplemented treatment remarkably increased, although non-significantly, the SOM content regarding the leonardite-alone treatment. It is important to note that this FSH-supplemented treatment contributed only 2–15% of soil increments of most macronutrients regarding the leonardite-alone, but remarkably higher increments of micronutrients (Table 7). These higher nutrient contributions by the leonardite-plus-FSH treatment were reflected in the plant-available soil P content, which featured a remarkable, though non-significant, increase regarding the leonardite-alone. However, this was reflected in the petioles P content, which featured a significant increase from the leonardite-alone treatment to the leonardite-plus-FSH one. Regarding K and the micronutrients, the increment of their plant-available soil contents from the leonardite-alone to the leonardite-plus-FSH treatment was also observed, although only significantly for Mn. Interestingly, the same trends of higher petiole contents of K and the micronutrients under the leonardite-plus-FSH treatment regarding the leonardite-alone treatment were observed.

The rise in the plant-available soil contents of all the aforementioned nutrients from the leonardite-alone to the leonardite-plus-FSH treatment can be explained on the basis of quantity, i.e., as indicated the leonardite-plus-FSH contributed higher nutrient amounts. However, the rise in the plant available soil contents under the leonardite FSH-supplemented treatment could be explained also on the basis of intensity. This would be due to the significant soil pH drop of 0.2 units under the leonardite-plus-FSH treatment.

Regarding the intensity effect of soil pH, this is recognized as the most relevant soil property in controlling element availability for plant nutrition [2,40]. Specifically, in the soil pH range 8.0–8.5, the plant-availability of P, Mn, Cu, and Zn increases as soil pH decreases [41], and it contributes to explaining the trend towards higher petiole contents for all these elements. Moreover, Ece et al. [42] also found a similar significant difference in plant P when applying leonardites to a slightly basic soil, low in SOM, and loamy-textured. Besides, both [23,43] also showed a better P crop nutritional status in their field trials with the combined application of sulphur and leonardites in corn and leonardite alone in cherry, respectively. The findings of these authors are thus in agreement with the ones obtained in the present work.

Therefore, the increased petiole P content in the present study suggests that both leonardite treatments could reduce soil P fixation while increasing its plant availability content, and the leonardite FSH-supplemented somewhat more than the leonardite-alone one. Therefore, since P is directly linked to the plant growth rate and energy transfer in plants, among other important metabolic processes [23], these leonardite treatments can alleviate the P deficiency in calcareous vineyard soils.

In order to understand why the leonardite-plus-FSH was able to significantly decrease soil pH, one can start from the fact Fe(II) in the ferrous sulphate heptahydrate is very labile in well-aerated high-pH soils [44] like the one in the study site. Under these conditions of well aeration and high soil pH, Fe(II) abiotically reacts with O_2_ to give Fe(III) which, in turn, experiences hydrolysis to precipitate as Fe(OH)_3_ while releasing 3 mols of H^+^ to the soil environment per each mol of precipitated Fe(III). The overall reaction is as follows:

(1) $$4{\mathrm{FeSO}}_{4}\cdot 7H_{2}O\left(s\right)+O_{2}\left(g\right)\to 4\mathrm{Fe}{\left(\mathrm{OH}\right)}_{3}\left(s\right)+4H_{2}{\mathrm{SO}}_{4}\left(\mathrm{aq}\right)+18H_{2}O\left(l\right)$$

where it is shown that one mol of sulphuric acid is released per each mol of ferrous sulphate heptahydrate. Once generated, the ferric hydroxide gradually evolves to form various ${\mathrm{Fe}}_{2}O_{3}\;\cdot {\;\mathrm{nH}}_{2}O$ solid species whereas the sulphuric acid is immediately neutralized by the soil ${\mathrm{CaCO}}_{3}$ according to the following equation:

(2) $$H_{2}{\mathrm{SO}}_{4}\left(\mathrm{aq}\right)+2{\mathrm{CaCO}}_{3}\left(s\right)\to 2{\mathrm{Ca}}^{2+}\left(\mathrm{aq}\right)+{\mathrm{SO}}_{4}^{2-}\left(\mathrm{aq}\right)+2{\mathrm{HCO}}_{3}^{-}\left(\mathrm{aq}\right)$$

Then, since hydrogen carbonate is a weak acid that dissociates according to the ensuing equation:

(3) $${\mathrm{HCO}}_{3}^{-}\left(\mathrm{aq}\right)↔{\mathrm{CO}}_{3}^{2-}\left(\mathrm{aq}\right)+H^{+}\left(\mathrm{aq}\right)$$

the fall in soil pH that results from the application of the leonardite FSH-supplemented amendment is at least qualitatively explained. Besides, the increase in the ion concentration of the soil solution because of ${\mathrm{Ca}}^{2+}$, ${\mathrm{SO}}_{4}^{2-}$, and ${\mathrm{HCO}}_{3}^{-}$ also qualitatively explains why the EC increases as a result of the leonardite FSH-supplemented treatment regarding the leonardite-alone one.

Beyond this qualitative analysis, however, the sulphuric acid that would have been recovered in a 1:2.5 soil:water suspension can be calculated from the rate and composition of FSH in the leonardite-plus-FSH amendment, as well as its method of application, and it results to be 0.05 mmol/L at most, i.e., disregarding the leaching due to the rainfall percolation during the growing season. This small amount would have only decreased the pH in 0.03 units regarding the purely calcareous soil and, concomitantly, it would have increased EC in only 8 µS/cm according to calculations carried out with the soil chemical speciation software SALSOLCHEMEC [45]. Therefore, these variations of −0.03 for pH and +8 µS/cm for EC do not compare well, in quantitative terms, to the observations of −0.2 for pH, and +100 µS/cm for EC.

These quantitative differences in soil pH and EC variations from the leonardite-alone to the leonardite-plus-FSH treatment between predictions and observations point towards another effect that is acting on the soil system under study in this work. In this regard, the leonardite-plus-FSH amendment rose the soil fertility status more than the leonardite-alone one because of both the quantity and intensity-soil pH-related effects that have been presented above. As a consequence, it can be hypothesized that the vine roots grew remarkably more under the leonardite-plus-FSH amendment than under the leonardite-alone one as a consequence of the higher nutrient availability in the leonardite-plus-FSH-amended soil part. This root growth thus contributed noticeably more fresh organic matter to the soil in the form of root exudates and lysates during the season under both leonardite treatments, but especially under the leonardite FSH-supplemented one. This proposed effect of mineral fertilization on SOM increasing is in accordance to the one that has been observed for paddies by [46].

Therefore, since a non-negligible part of the organic rhizodeposition by higher plants is in the form of low-molecular-weight acids [47], and since the higher activity and thus respiration rate of better-fed soil organisms very likely released more simple organic acids, carbon dioxide, and inorganic ions to the soil solution, then soil pH decreased and EC increased much more than expected on the basis of the first purely abiotic effect depicted by Equations (1)–(3). In this regard, the FSH can be thought of as acting through such an abiotic effect as a primer for the SOM dynamics in this SOM- and nutrient-impoverished soil. Notwithstanding, it should be also noted that the shaking time over 10 min, and non-centrifugation of the 1: 2.5 soil: water suspensions could have somewhat contributed to exaggerate the soil pH and EC measurements due to the higher lysis of microbial and sloughed-off plant cells under such analytical conditions as pointed out in [47].

The concomitant increase in the iron plant-available soil contents in the present study under the leonardite-plus-FSH treatment can therefore be explained on the basis of the increased SOM content and dynamics. This is because the soil biological activity fosters the Fe(III) reduction to Fe(II) in micropores where organic matter respiration depletes the $O_{2}$ concentration [48], and additionally because of the likely binding of Fe(III) to deprotonated simple organic acids [47]. This effect on available Fe seems to have been extended to Mn and Zn, and the beneficial effects of mixing leonardite with FSH for improving the soil micronutrients plant-availability makes this a better practice than using leonardite alone, overall to replace iron chelates, which is a common treatment to alleviate Fe plant-deficiency in calcareous soils [36]. In terms of future research, studies to assess the short- and long-term effectiveness of leonardite treatments on soil fertility conditions would be interesting from such an iron chlorosis point of view. In this regard, the likely non-easily leached iron from the leonardite-plus-FSH amendment could be an advantage over Fe chelates because these are easily leached in rainy springs or in irrigated systems [49] and, consequently, they usually have to be applied once or twice a year [50].

Regarding the harvest quality, none of the leonardite treatments significantly affected the grape must quality parameters, with the sole exception of must pH. In spite of this fact, however, a noticeable trend towards increased K in soil and in vine petioles, as well as in grape musts, in response to the leonardite treatments, with a concomitant decrease in real and total acidity of grape must, was observed, and these trends were most apparent in the leonardite-plus-FSH treatment. In this regard, Ece et al. [42] also noted higher soil K in leonardite-amended plots when compared to control ones. This is an important subject for future research in wine production because an increase in grape must pH implies obtaining wines with lack of colour [51] and freshness, and which age faster than intended, as well as dampening the antimicrobial effect of ${\mathrm{SO}}_{2}$ [52].

Additionally, another increasing trend was observed in total soluble solids as a consequence of the leonardite treatments. This finding is consistent with that reported by Ramos and Romero [51], who observed how total soluble solids were positively correlated with K in grape flesh. However, this is contrary to the results obtained by Ciotta et al. [53], who demonstrated that an increase of exchangeable K in a sandy soil increased K in leaves and berries, as well as grape must pH, but on the other hand, it promoted lower total soluble solids. Moreover, because the synthesis and accumulation of aromatic compounds and polyphenols are modified by nutritional Fe deficiency [54], future studies on this topic are recommended to assess the effects of leonardite amendments on all these important harvest parameters for wine production.

The findings of the research here reported should be interpreted with caution since statistical power was not high enough and hence, in general, little significance was achieved, and since some important soil parameters were not measured, e.g., root mass fractions in the soil under the control and the different treatments, as well as berry yield. Whatever the case, leonardite seems to behave as a beneficial organic amendment for improving the soil fertility in calcareous vineyard soils in the context of ecological/sustainable viticulture systems, and its effects seem to severely increase when used in conjunction with another amendment material able to provide nutrients while at the same time fostering a better soil pH for plant development. Therefore, leonardite seems to have potential as an alternative to other organic amendment materials and FSH-supplemented as an alternative to synthetic nutrient chelates for plant nutrition. Further studies are needed to assess the effects of leonardite on other vineyard soil types as well as grape varieties and rootstocks.

## 4. Materials and Methods

### 4.1. Study Site

**Figure 9:**
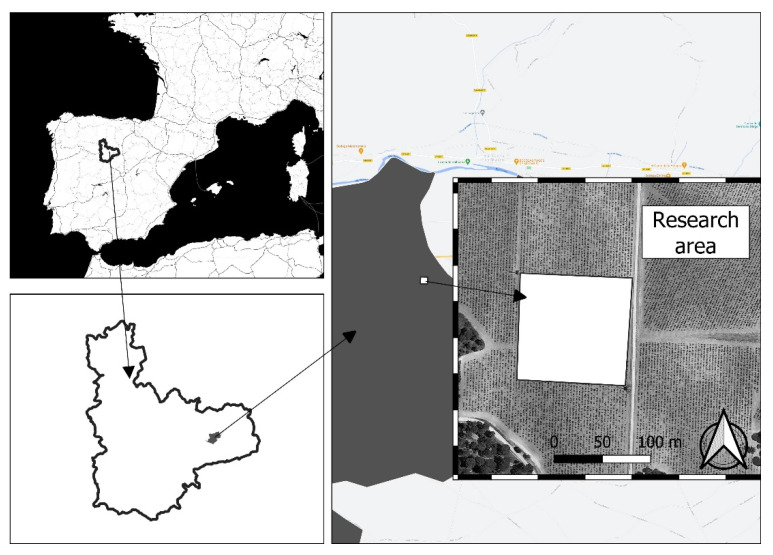
Location map of the research area within the municipality of Valbuena de Duero in grey, and this within the Valladolid province surrounded by the solid line in Spain.

The study site was a commercial vineyard located approximately 750 m above the sea level within the protected designation of origin (PDO) Ribera del Duero in the municipality of Valbuena de Duero in Valladolid, Spain, at latitude 41°37′ N and longitude 4°18′ W (see Figure 9). The climate in the site is classified as warm-summer Mediterranean (Csb) following the Köppen classification, with an aridity index between 0.65 and 0.80 according to UNEP-FAO classification. During the years 2017–2019, the weather was characterised by average temperature of, respectively, 12.7, 12.1, and 12.2 °C, cumulative reference evapotranspiration (FAO Penman-Monteith) of 1190, 1040, and 1130 mm, and cumulative rainfall of 273, 664, and 427 mm [49]. The soil under study corresponds to a Calcareous cambisol according to FAO’s reference base, which showed a clay loam textural class and a strong effervescence to HCl in its entire profile, whereas the parent material of the soils in the study area comprises Tertiary materials from the Miocene.

The research was conducted on about 35-year-old *Vitis vinifera* L. cv. “Tempranillo” grapevines grafted onto 41-B rootstocks. Rows were north–south oriented, and 3.0 m spaced with 1.5 m between plants. Vines were head-trained with 3–4 arms, where nodes were retained at winter with guyot mixed pruning, leaving one spur with two buds and one fruiting shoot with six buds per arm. The vineyard had no irrigation system support, and no fertilizers or extra amendments other than those used in this research were applied to the soil under study.

### 4.2. Characterization of the Fertilizer Materials and Doses

The composition on a dry-matter basis of the leonardite and FSH materials that were used in this study is shown in Table 8. The pH, and electrical conductivity at 25 °C (EC) were measured in 1: 5 solid: water (*w*/*v*) suspensions using, respectively, a micropH 2001 pHmeter and an EC conductimeter 522 (CRISON, Barcelona, Spain). Total N was determined by means of the Kjeldahl method using a KjelFlex K-360 steam distillation unit (BUCHI, Flawil, Switzerland). Phosphorus (P) was measured by ultraviolet-visible spectroscopy (libra, Biochrom, Cambridge, UK) after microwave digestion using a Multiwave GOPlus oven (Anton Paar, Graz, Austria). Finally, Ca, Mg, K, Fe, Mn, Cu, and Zn were measured by inductively coupled plasma atomic emission spectroscopy (ICP-AES) using an iCAP 7000 series (ThermoFisher Scientific, Waltham, MA, USA) after microwave digestion. As additional information regarding leonardite (according to the manufacturer SEPHU©), 20% of its organic matter consisted of humic substances (both humic and fulvic acids).

The pH and EC of 1:1, 2:1, 3:1, and 4:1 weight mixtures (w:w) of leonardite and FSH in mixture:water (1:5) were measured (see Table 9). According to all these characteristics, it was decided to apply the following treatment doses: 1000 kg/ha of CL and 1500 kg/ha of LS (≈15 and 50% decrease in pH and EC respectively of weight mixture 2:1 with respect to the leonardite treatment alone). It should be noted that the leonardite dose used in this experiment was, on average, the leonardite rate that is traditionally used by the vine growers in the calcareous soil vineyards from this region.

### 4.3. Experimental Design

Three treatments, with three replications per treatment, were applied: C, CL, and LS. The study plot was split into 9 subplots with eight vines in each (with two buffer vines, and one buffer row between subplots). Because of the homogeneity of the soil area under study (about 600 m^2^), the treatment replications were distributed among the 9 subplots (about 31.5 m^2^ each) in a completely random design with three treatments per row.

The treatments were applied on 30-cm wide bands along the plantation lanes about 0.5 m from the grapevine line, with subsequent manual incorporation, at a depth of approximately 0.25 m every year. In 2017, the amendments were applied at green tip (number 4 of modified Eichorn-Lorenz (E-L) system), whereas in 2018 and 2019 they were at winter bud (number 1 of modified E-L system) [6]. The application side of the treatments changed alternatively each year.

### 4.4. Soil Sampling and Analyses

Before the amendments were applied, an agronomic characterisation of the calcareous vineyard soil at depths of 0–30 and 30–60 cm was carried out based on the following soil properties: texture, SOM, soil pH in water (pHw), electrical conductivity (EC), total and active carbonates, phosphorus (P) and potassium (K) contents. In addition, the following micronutrients were considered: iron (Fe), copper (Cu), manganese (Mn) and zinc (Zn). The effects of the treatments on the following soil properties in each subplot were monitored for three years (2017, 2018 and 2019): pH, EC, SOM, nitrogen (N), organic carbon, C/N ratio, and plant-available P, K, Fe, Mn, Cu and Zn contents. This monitoring was conducted by sampling the soil at a depth of 0–30 cm at the senescence phenological stage (end of leaf fall).

The soil samples (before and after the amendment application) were collected by drilling with an auger in spots along the bands where the amendments had been applied. Afterwards, they were sealed in plastic bags, transported to the laboratory, and air-dried at room temperature. Next, they were gently disaggregated, passed through a 2-mm mesh sieve, and adequately stored until analysis. The texture according to the USDA was determined by the hydrometer method. The organic carbon and, subsequently, the SOM, were determined by Walkley–Black wet oxidation. The pH and EC were measured in the supernatants of soil: water 1: 2.5 suspensions following 25 min of shaking and 5 min of soil settlement, and using, respectively, a micropH 2001 pHmeter and a conductimeter 522. Soil nitrogen was determined by the Kjeldahl method [55], which determines all soil nitrogen except that in nitrates [56]. Soil phosphorus available for vines was determined by ultraviolet-visible spectroscopy after treatment with the Olsen–Watanabe extractant. Exchangeable potassium was determined by extraction with successive aliquots of 1 M ammonium acetate (${\mathrm{NH}}_{4}C_{2}H_{3}O_{2}$) followed by determination by atomic absorption spectrometry (AAS; Unicam SOLAAR 969). The micronutrients (Fe, Cu, Mn, and Zn) were extracted following the ${\mathrm{CaCl}}_{2}\mathrm{DTPA}$ method (which correlates well with Mehlich-3 extraction and is well established for calcareous soils [57,58]) using a buffer solution of 0.005 M diethylenetriaminepentaacetic acid (DTPA) and 0.01 M calcium chloride (${\mathrm{CaCl}}_{2}$) buffered to pH 7.3 with subsequent determination in the extracts by AAS (Unicam SOLAAR 969). The total carbonates and active carbonates were determined with the Bernard calcimeter method, after extraction with ammonium oxalate 0.2 N for the last ones.

### 4.5. Initial Soil Characterisation before Application of the Treatments

Table 10 shows the baseline characteristics of the calcareous soil under study (at 0–30 and 30–60 cm depths) before application of the treatments. In Table 1, it can be observed that the soil was poor in organic matter (<1.5% at both topsoil and subsoil), with clay dominant throughout its depth. The soil pH values were above 8.0, and the total carbonates were high, whereas active carbonates could be considered as moderate. The presence of carbonates (both total and active) causes common grape production problems which include low P and micronutrient availability. However, the soil showed a reasonable good P level (especially at 0–30 cm depth), but low Fe and Zn levels [58].

### 4.6. Leaf Sampling and Analyses

Like the soil properties, the P, K, Fe, Mn, Cu, and Zn contents in petioles (P-p, K-p, Fe-p, Mn-p, Cu-p and Zn-p respectively) were determined annually, but at the veraison phenological stage. Specifically, around 20 grape basal leaves located in opposite bunches were randomly collected per subplot each year. They were sealed in paper bags and transported to the laboratory. The leaves were carefully rinsed with abundant deionised water, and then the petioles were cut and dried for three days at 70 °C [59]. Next, they were wet-digested with an acidic mixture of perchloric, sulphuric and nitric acids at 420 °C for 20 min, and the element contents in the extracts were determined by ICP-AES (iCAP 7000 series, ThermoFisher Scientific, Waltham, MA, USA).

### 4.7. Grape Sampling and Analyses

Like soil and petioles, the grapes were sampled at harvest each year (during the second half of September), when degrees Brix readings taken in the vineyard were, at least, 22 °Bx. In each subplot, 100 grape berries were randomly chosen to determine their weight (W) and the grape must quality parameters. The grape must from each subplot was obtained manually from the 100 berries by gently pressing the berries and using rubber gloves to avoid contamination. The following harvest quality properties of the grape must were determined: (i) real acidity (pH) with a CRISON micropH 2001 (Spain, Barcelona), (ii) total soluble solids (Brix) using a refractometer (Zuzi Series 300, Auxilab, Spain), (iii) titratable acidity (TA) by titration of the grape must with sodium hydroxide (0.1 M) to an endpoint of pH 7.0, and expressed as the equivalent content of tartaric acid in g/L, (iv) potassium level (K-m) in mg/L by analysis of its content by direct reading in atomic absorption spectrometry (AAS) with SOLAAR 969 (Thermo Spectronics Unicam, Waltham, MA, USA), (v) malic acid (MA) and tartaric acid (TcA) at 340 and 492 nm wavelength, respectively, and (vi) yeast assimilable nitrogen (YAN) from the primary amino acids and ammonium ion, by enzymatic methods (Analyzer BA400, BioSystems, Spain, Barcelona).

### 4.8. Statistical Analyses

Statistical analyses were performed using the R software [60]. Several ANOVAs were carried out to study the effects of the leonardite treatments on (i) soil chemical properties, (ii) petiole nutrient contents, and (iii) grape must quality properties. Previously, an identification of potential outliers was performed.

Mixed ANOVAs were used to determine whether the differences between treatments (T) were statistically significant and whether they depended on the year of sampling (Y), and to determine the interaction between both factors. In this mixed design, the treatment factor was set as the between-group predictor and the year factor was used as a random factor. To reveal the overall effect of each main effect and its interactions, a hierarchical multilevel model approach was used, in which models were built up with one predictor at a time from a baseline with no predictors other than the intercept. Factors in these nested models were added in the following order: no predictors, T, Y, and the interaction between T and Y. Maximum likelihood (ML) was used to compare the nested models using a variance analysis.

If the interaction between factors resulted in a significant effect, we did not interpret any main effects, because the higher-order interaction superseded it. In that case, the effect of treatment was split independently for each year of the research and studied using orthogonal comparisons to determine which groups differed significantly. On the other hand, if the interaction between factors did not show any significant effect, the main effect of treatment dose was interpreted independently of the factor ‘year’, while the main effect of the factor ‘year’ was ignored. When sample sizes are equal, mixed ANOVA, is quite and fairly robust, respectively, in terms of the error rate associated with violations of normality and assumption of homogeneity of variance (homoscedasticity) [61]. This was the situation in the present study.

When the likelihood ratio of mixed ANOVAs was large enough to be statistically significant, orthogonal comparisons were carried out to determine which groups significantly differed (at the * *p* < 0.05, ** *p* < 0.01 or *** *p* < 0.001 level). These comparisons were performed with specific hypotheses. Thus, a partitioned experimental variance into component comparisons (in each contrast, there were compared only two chunks of variance) was performed: contrast 1 (C1) involved comparing the control group to both treatment groups (C vs. CL + LS), whereas contrast 2 (C2) involved comparing one treatment group to the other (CL vs. LS).

## 5. Conclusions

The leonardite treatments increased the soil organic matter and, in general, improved the plant-availability of soil nutrients in a calcareous soil cultivated with *Vitis vinifera* L. cv. Tempranillo. A relevant soil acidification effect was further observed when leonardite was mixed with ferrous sulphate heptahydrate, which additionally encouraged the soil plant-availability of phosphorus as well as the other nutrients. The leonardite and the FSH-supplemented one seemed to have potential to foster the vine root growth in the soil spots where it had been applied because of nutrient quantity and intensity-pH-related effects and, therefore, to trigger SOM dynamics and build-up. This primer effect would explain the further beneficial effects of the leonardite-plus-FSH amendment on plant-available nutrient contents.

Notwithstanding the limitations of the research regarding statistical significance and lack of measurement of some variables, importantly, root mass fraction in the soil under the various treatments, the results obtained provide evidences for the potential use of leonardite as an alternative amendment to other organic materials such as manures in calcareous vineyard soils. This is particularly important to avoid a common problem of soil pollution induced by animal manures, which often act as sources of heavy metals and other pollutants.

Finally, our research did not detect any statistically significant evidence of effects on berry weight and harvest quality as a result of the leonardite treatments, even though a tendency towards a higher translocation of potassium from soil to plant, and to grape, could be observed as a consequence of the leonardite treatments.

## Figures and Tables

**Table 1:** Analysis of variance (ML: Maximum likelihood) performed on the soil parameters (pH, electrical conductivity (EC), soil organic matter (SOM), nitrogen (N), C/N ratio, phosphorus (P), potassium (K), iron (Fe), manganese (Mn), copper (Cu) and zinc (Zn)) at the leaf fall stage. The results were significant at * *p* < 0.05, ** *p* < 0.01 and *** *p* < 0.01.

Soil Parameter	ML Ratio (Treatment (T))	ML Ratio (Year (Y))	ML Ratio (T × Y)
pH	7.68 (*)	8.39 (*)	3.97
EC	3.03	2.02	3.38
SOM	8.76 (*)	8.28 (*)	0.35
N	5.44	8.75 (*)	21.2 (***)
C/N	0.51	3.10	37.8 (***)
P	3.63	4.88	1.86
K	5.69	0.11	0.67
Fe	3.93	6.66 (*)	2.32
Mn	8.08 (*)	13.1 (**)	1.74
Cu	1.38	7.37 (*)	3.68
Zn	7.13 (*)	12.5 (**)	5.17

**Table 2:** Mean differences in planned contrasts (C1 and C2) on the soil parameters (pH, soil organic matter (SOM), manganese (Mn) and zinc (Zn)) where the treatments had a significant effect. The results were significant at * *p* < 0.05.

Soil Parameter	C1	C2
pH	−0.03 (*)	−0.10 (*)
SOM	0.09 (*)	0.14
Mn	0.21	0.62 (*)
Zn	0.04 (*)	0.05

**Table 3:** Analysis of variance (ML: Maximum likelihood) performed on petiole nutrient contents (phosphorus (P-p), potassium (K-p), iron (Fe-p), manganese (Mn-p), copper (Cu-p) and zinc (Zn-p)) at the veraison stage. The results were significant at * *p* < 0.05, ** *p* < 0.01 and *** *p* < 0.001.

Petiole Nutrient	ML Ratio (Treatment (T))	ML Ratio (Year (Y))	ML Ratio (T × Y)
P-p	7.20 (*)	9.62 (**)	3.71
K-p	2.18	14.0 (***)	3.72
Fe-p	3.00	9.06 (*)	1.31
Mn-p	0.52	1.83	5.65
Cu-p	1.32	9.61 (**)	2.69
Zn-p	3.26	19.2 (***)	3.84

**Table 4:** Mean differences in planned contrasts (C1 and C2) on the petiole phosphorus content (P-p). The results were significant at * *p* < 0.05.

Petiole Nutrient	C1	C2
P-p	0.01 (*)	0.01

**Table 5:** Analysis of variance (ML: Maximum likelihood) performed on harvest parameters (weight of 100 grape berries (W100), real acidity (pH), titratable acidity (TA), Brix degrees (Brix), malic acid (MA), tartaric acid (TcA), must potassium levels (K-m) and yeast assimilable nitrogen (YAN)). The results were significant at * *p* < 0.05, ** *p* < 0.01 and *** *p* < 0.001.

Harvest Parameter	ML Ratio (Treatment (T))	ML Ratio (Year (Y))	ML Ratio (T × Y)
W100	2.06	14.0 (***)	3.20
pH	7.15 (*)	13.9 (**)	0.92
TA	1.49	2.03	5.48
Brix	5.40	1.92	1.66
MA	2.73	0.93	2.93
TcA	0.95	3.74	1.25
K-m	0.76	13.2 (**)	4.25
YAN	0.74	14.1 (***)	0.63

**Table 6:** Mean differences in planned contrasts (C1 and C2) on harvest parameter real acidity (pH). The results were significant at * *p* < 0.05.

Harvest Parameter	C1	C2
pH	0.02 (*)	−0.01

**Table 7:** Maximum increments of SOM and nutrients expected in the leonardite-amended soils according to the amendments’ rates, compositions and way of application (30-cm wide band application down to 30 cm depth, and soil bulk density of 1.5 g/cm^3^). All increments in mg/kg except for SOM which is in %.

Amendment	ΔSOM	ΔN	ΔP	ΔK	ΔFe	ΔMn	ΔCu	ΔZn
CL	0.064	3.8	5.6	6.0	36	0.12	0.09	0.06
LS	0.067	4.3	6.0	6.1	163	3.0	0.15	0.41

**Table 8:** Characteristics of the leonardite and ferrous sulphate heptahydrate (FSH) materials expressed on a dry-matter basis. ^a^ Electrical conductivity (EC) in dS/m; ^b^ organic matter (OM), N, P, Ca, Mg, K, and Fe in %; ^c^ Mn, Cu, and Zn in mg/kg (n = 3).

Amendment Material	pH	EC ^a^	OM ^b^	N ^b^	P ^b^	Ca ^b^	Mg ^b^	K ^b^	Fe ^b^	Mn ^c^	Cu ^c^	Zn ^c^
Leonardite	2.52	6.42	28.8	0.17	0.25	0.80	0.08	0.27	1.64	55.9	40.6	25.9
FSH	2.02	22.9	2.94	0.05	0.04	0.06	0.26	0.01	11.4	2620	51.7	320

**Table 9:** pH and electrical conductivity (EC; in dS/m) of mixtures of leonardite and ferrous sulphate heptahydrate (FSH) (in weight:weight ratio (w:w)) in mixture-to-water suspensions (1:5) (n = 3).

Leonardite:FSH (w:w)	pH	EC
1:1	2.32	16.7
2:1	2.38	12.1
3:1	2.44	10.7
4:1	2.50	9.70

**Table 10:** Average soil characteristics before application of the treatments (n = 3).

Soil Parameter	0–30 cm	30–60 cm
Sand (%)	44.7	40.7
Silt (%)	13.9	9.9
Clay (%)	41.4	49.4
Textural class (USDA)	Clay	Clay
pH	8.26	8.27
EC (dS/m)	0.17	0.18
SOM (%)	1.27	1.12
Total carbonates (%)	39.2	37.7
Active carbonates (%)	9.00	10.8
P (mg/kg)	22.3	9.67
K (cmol(+)/kg)	1.19	0.96
Fe (mg/kg)	6.65	6.43
Mn (mg/kg)	6.00	6.74
Cu (mg/kg)	1.29	0.96
Zn (mg/kg)	0.60	0.23

## Data Availability

Not applicable.

## Abbreviations

Aqueous (aq); analysis of variance (ANOVA); atomic absorption spectrometry (AAS); contrast 1 (C1); contrast 2 (C2); control not treated (C); control of leonardite (CL); electrical conductivity (EC); inductively coupled plasma atomic emission spectroscopy (ICP-AES); modified Eichorn-Lorenz system (E-L); ferrous sulphate heptahydrate (FSH); leonardite mixed with ferrous sulphate heptahydrate (LS); gas (g); grape must malic acid (MA); maximum likelihood (ML); grape must potassium level (K-m); grape must tartaric acid (TcA); grape must titratable acidity (TA); grape must yeast assimilable nitrogen (YAN); oceanic with dry summer (Csb); petiole phosphorus, potassium, iron, copper, manganese and zinc content (P-p, K-p, Fe-p, Mn-p, Cu-p and Zn-p respectively); protected designation of origin (PDO); soil organic matter (SOM); solid (s); treatment (T); year of sampling (Y); soil pH (pH); soil phosphorus, potassium, iron, copper, manganese and zinc content (P, K, Fe, Cu, Mn and Zn respectively); Weight of 100 grape berries (W100).
